# Optical Activation of TrkB (E281A) in Excitatory and Inhibitory Neurons of the Mouse Visual Cortex

**DOI:** 10.3390/ijms231810249

**Published:** 2022-09-06

**Authors:** Antonia Lilja, Giuliano Didio, Jongryul Hong, Won Do Heo, Eero Castrén, Juzoh Umemori

**Affiliations:** 1 Faculty of Psychology and Neuroscience, Maastricht University, 6229 ER Maastricht, The Netherlands; 2 Neuroscience Center, Helsinki Institute of Life Science (HiLIFE), University of Helsinki, 00100 Helsinki, Finland; 3 Department of Biological Science, Korea Advanced Institute of Science and Technology (KAIST), Daejeon 305-701, Korea; 4 Center for Cognition and Sociality, Institute for Basic Science (IBS), Daejeon 305-701, Korea; 5 KAIST Institute for the BioCentury, KAIST, Daejeon 305-701, Korea; 6 Gene and Cell Technology, A.I. Virtanen Institute, University of Eastern Finland, 70211 Kuopio, Finland

**Keywords:** neural plasticity, tropomyosin kinase receptor B, parvalbumin-positive interneurons, calcium/calmodulin positive pyramidal neurons, V1/visual cortex, multiple regression with interaction and simple slopes analysis

## Abstract

The activation of tropomyosin receptor kinase B (TrkB), the receptor of brain-derived neurotrophic factor (BDNF), plays a key role in induced juvenile-like plasticity (iPlasticity), which allows restructuring of neural networks in adulthood. Optically activatable TrkB (optoTrkB) can temporarily and spatially evoke iPlasticity, and recently, optoTrkB (E281A) was developed as a variant that is highly sensitive to light stimulation while having lower basal activity compared to the original optoTrkB. In this study, we validate optoTrkB (E281A) activated in alpha calcium/calmodulin-dependent protein kinase type II positive (CKII^+^) pyramidal neurons or parvalbumin-positive (PV^+^) interneurons in the mouse visual cortex by immunohistochemistry. OptoTrkB (E281A) was activated in PV^+^ interneurons and CKII^+^ pyramidal neurons with blue light (488 nm) through the intact skull and fur, and through a transparent skull, respectively. LED light stimulation significantly increased the intensity of phosphorylated ERK and CREB even through intact skull and fur. These findings indicate that the highly sensitive optoTrkB (E281A) can be used in iPlasticity studies of both inhibitory and excitatory neurons, with flexible stimulation protocols in behavioural studies.

## 1. Introduction

Neural plasticity refers to the processes through which environmental factors influence neuronal structures and functions [1]. Two key players in neural plasticity are brain-derived neurotrophic factor (BDNF) and its high-affinity receptor, tropomyosin receptor kinase B (TrkB), which supports the development of neurons and neuroplasticity at a cellular, synaptic, and network level. When BDNF binds to TrkB, two TrkB receptors form a dimer that autophosphorylates [2]. This sets in motion several signalling cascades, including induction of long term potentiation (LTP), which is crucial for memory and learning [2].

After a critical period of heightened neural plasticity in childhood [3,4], neural networks become consolidated and neurotrophin signalling patterns change [5,6,7]. However, various interventions, such as antidepressant treatment, have been shown to induce a state of increased neuroplasticity in the adult brain that is similar to that of the juvenile critical period [1,8]. This artificially induced juvenile-like neuroplasticity in the adult brain has been coined iPlasticity [1,9].

iPlasticity has frequently been studied in the visual cortex. Eye-specific ocular dominance develops in the visual cortex during the juvenile critical period [4]. In adult age, this ocular dominance can be changed by restoring visual cortex plasticity. This can be done using antidepressants, such as fluoxetine. Chronic administration of fluoxetine has been shown to successfully induce amblyopia, shift ocular dominance, and increase levels of BDNF in the visual cortex [10]. Further insight into the reinstatement of visual cortex plasticity has been provided by studies investigating parvalbumin-positive (PV^+^) interneurons. Our group recently showed that fluoxetine failed to induce visual cortex plasticity when PV^+^ interneurons expressed a reduced amount of TrkB [11].

Optically activatable TrkB (optoTrkB) has been developed to study the effects of TrkB activation with high spatiotemporal specificity [12]. In optoTrkB, a photolyase homology region (PHR) of cryptochrome 2, a blue light receptor, is fused with a TrkB receptor, and blue light stimulation successfully activates TrkB signalling pathways [12]. OptoTrkB is preferable to other approaches to manipulate TrkB signalling, since optoTrkB can be activated in specific cells and brain areas at specific times. This allows us to establish causal relationships between TrkB signalling, neural network changes, and behavioural effects with temporal and spatial precision [13]. Recently, a new type of optoTrkB (E281A) was developed and successfully activated by blue light stimulation through the intact skull and fur of mice [13]. OptoTrkB (E281A) also showed a low level of basal activity, allowing for more experimental control as spontaneous optoTrkB signalling was reduced. OptoTrkB (E281A) was successfully activated and the phosphorylation of extracellular signal-regulated kinase (pERK) was increased in CKII^+^ neurons of the dentate gyrus in the hippocampus. However, it is unknown if optoTrkB (E281A) can be activated in other neuronal subpopulations than CKII^+^ pyramidal neurons and in other brain regions than in the hippocampus.

OptoTrkB is introduced in a double-inverted open reading frame structure (DIO), whereby the optoTrkB sequence is inverted and flanked by two incompatible lox-P sites. This structure allows to turn on TrkB expression via Cre recombinase-mediated recombination [14,15]. Thus, optoTrkB can be expressed in a specific neuronal subpopulation by injecting it into a certain region of genetically modified mice expressing Cre recombinase in specific cells, such as CKII- and PV- positive neurons. Adeno-associated virus (AAV) is one of the most widely used viral vehicles of genetic information and has lower immunogenicity than other viruses, making it less likely to be eliminated by the immune system before gene delivery [16].

**Figure 1:**
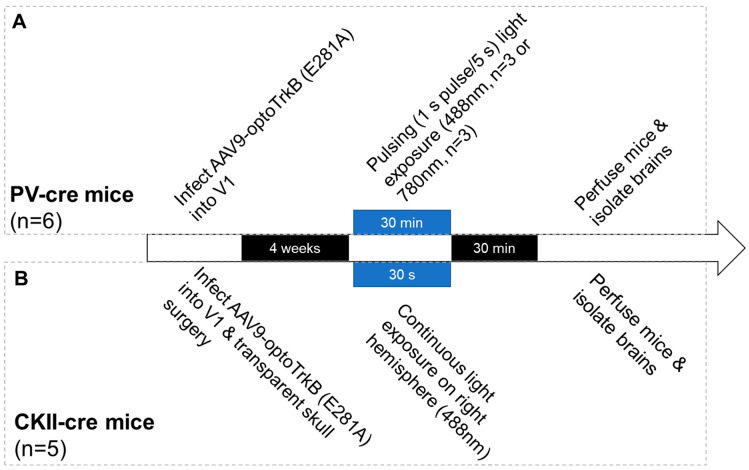
Timeline of optoTrkB (E281) stimulation and perfusion. (**A**) Stimulation protocol for PV-Cre mice. (**B**) Stimulation protocol for CKII-Cre mice.

Thus, in this study, we infected AAV-DIO-optoTrkB (E281A) into the visual cortex of mice lines that express Cre recombinase specifically in CKII^+^ pyramidal neurons and PV^+^ interneurons, in order to express optoTrkB in the specific neuronal subpopulations (Figure 1). OptoTrkB (E281A) activation was then triggered with blue light stimulation through the transparent skull or atop the intact fur head. Then, we validated the activation by immunohistochemistry of two TrkB downstream signals, pERK and cAMP-calcium response element binding protein (CREB), a transcription factor that plays a key role in LTP [2].

## 2. Results

### 2.1. Activation of OptoTrkB (E281A) in PV^+^ Interneurons

**Figure 2:**
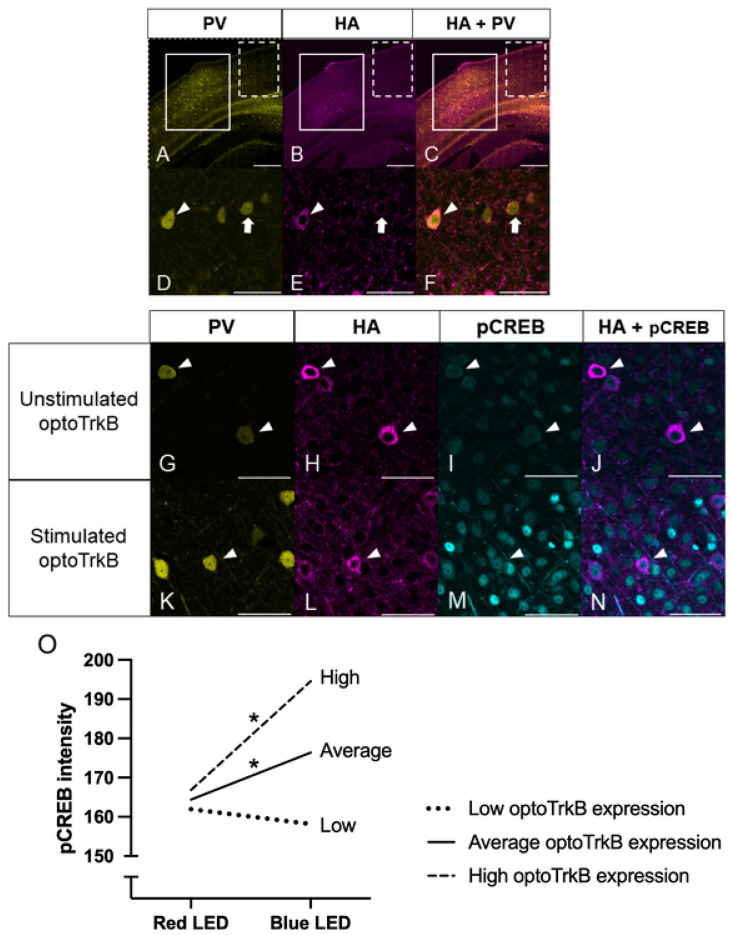
(**A**–**F**) OptoTrkB (E281A) was successfully expressed and activated in PV^+^ interneurons in the visual cortex of PV-Cre mice. Representative images of V1 stained with anti-PV antibody (**A**,**D**), HA antibody (**B**,**E**), and merged (**C**,**F**). HA-expressing neurons also express PV, indicating that optoTrkB (E281A) is specifically expressed in PV^+^ interneurons. Solid outline, dashed outline, arrowheads, and arrows indicate the infected area, non-infected area, PV^+^ interneurons infected with optoTrkB (E281A), and PV^+^ interneurons not infected with optoTrkB (E281A), respectively. (**G**–**N**) Light-stimulated PV^+^ interneurons show higher pCREB intensity than unstimulated PV^+^ interneurons. Representative images of V1 staining with antibodies of anti-PV (**G**,**K**), HA (**H**,**L**), pCREB (**I**,**M**), and merged HA and pCREB (**J**,**N**). Neurons in unstimulated (**G**–**J**), and stimulated brain (**K**–**N**). Scale bar (**A**–**C**): 500 um; scale bar (**D**–**N**): 50 um. Arrowheads point to PV^+^ interneurons infected with optoTrkB. (**O**) Regression slopes for the effect of LED light stimulation on pCREB for high (dash line), average (solid line), and low (dot line) levels of optoTrkB (E281A). OptoTrkB (E281A) stimulation significantly increased pCREB intensity for neurons expressing high and average levels of optoTrkB (E281A), but not for those with low expression levels of optoTrkB (E281A). The regression equations for cells expressing optoTrkB (E281A) at high, average, and low level are as follows: pCREB high HA=166.839+27.722∗stimulation+0.040∗HAintensity+0.257∗stimulation∗HAintensity+error (p (β_stimulation_) = 0.001); pCREB average HA=164.402+11.962∗stimulation+0.040∗HAintensity+0.257∗stimulation∗HAintensity+error (p (β_stimulation_) = 0.007); pCREB low HA=161.956−3.798∗stimulation+0.040∗HAintensity+0.257∗stimulation∗HAintensity+error (p (β_stimulation_) = 0.284). * *p* < 0.017 (Bonferroni-corrected threshold).

Expression of optoTrkB (E281A) can be inferred by the intensity of human influenza hemagglutinin (HA) tagged at the C-terminal of optoTrkB. Immunohistochemistry by co-staining with anti-PV and anti-HA antibodies in PV-Cre mice infected with a pAAV-hSyn1-DIO-OptocytTrkB(E281A)-HA construct showed that HA-positive neurons overlapped with PV^+^ interneurons, indicating successful infection of the visual cortex in PV-Cre mice (Figure 2A–F). Closer inspection of individual neurons showed that HA staining surrounded PV-stained cells (Figure 2D–F), indicating that optoTrkB (E281A) was successfully infected in PV^+^ interneurons of PV-Cre mice.

Image analysis revealed that cells in the infected area of PV-Cre mice expressed HA more strongly than in the uninfected area (Figure 2A–C). However, anti-HA partially stains background particles, and there are also some non-infected PV^+^ interneurons in the infected area. To ensure that only neurons infected with optoTrkB (E281A) tagged with HA were included for further analysis, a cutoff point for minimum HA intensity was set by comparing the distribution of HA intensity values in the cells from the infected area (N = 88) to those of an uninfected adjacent area (N = 60). The point of overlap of these distribution curves was selected as the cutoff point for minimum HA intensity (Figure S1). Neurons from the infected area that had lower HA intensity than this cutoff were excluded from further analysis (Figure S2). After applying this cutoff value, 44 stimulated neurons and 41 unstimulated neurons remained for the final analysis.

The intensity of pCREB was analysed in the cells expressing optoTrkB (E281) (Figure 2G–N). A multiple regression assessing the effect of optoTrkB (E281A) stimulation and HA intensity on pCREB intensity revealed a significant difference in pCREB between the groups (F(3, 81) = 5.414, *p* = 0.002). Inspection of the regression coefficients revealed a significant interaction effect between stimulation and HA intensity (β = 0.257, *p* = 0.004), suggesting that the effect of optoTrkB (E281A) stimulation on downstream signalling depends on the extent to which the neuron expressed optoTrkB (E281A) receptors. Simple slope analysis was then conducted by analysing the effect of optoTrkB (E281A) stimulation at average, low (one standard deviation below the average), and high (one standard deviation above the average) optoTrkB (E281A) intensity (Figure 2O). The analysis showed that at average HA intensity, optoTrkB (E281A) stimulation significantly increased pCREB intensity (β = 11.962, *p* = 0.007). Similarly, at one standard deviation above average HA intensity, optoTrkB (E281A) stimulation significantly increased pCREB intensity (β = 27.722, *p* = 0.001). However, at one standard deviation below average HA intensity, there was no effect of optoTrkB (E281A) stimulation on pCREB intensity (β = 3.798, *p* = 0.506). These results indicate that pCREB increased after optoTrkB stimulation only in PV^+^ interneurons expressing average to high levels of optoTrkB (E281A).

### 2.2. Activation of OptoTrkB (E281A) in CKII^+^ Pyramidal Neurons

**Figure 3:**
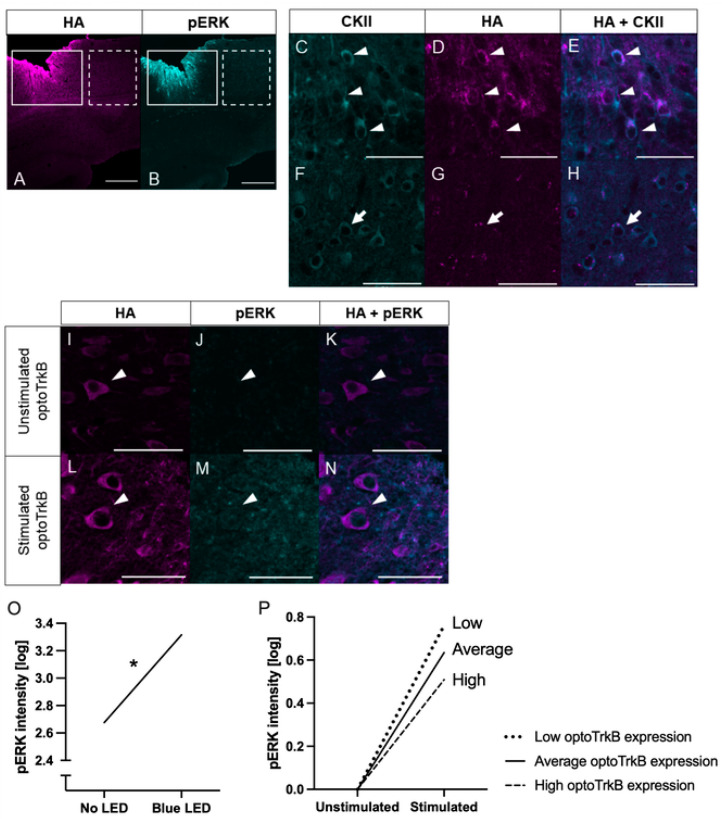
(**A**–**H**) OptoTrkB (E281A) was successfully expressed in CKII^+^ neurons in the visual cortex of CKII-Cre mice. Representative images of V1 stained with anti-CKII antibody (**C**,**F**), HA antibody (**A**,**D**,**G**), pERK antibody (**B**), and merged CKII and HA (**E**,**H**). HA expressing neurons also express CKII, indicating that optoTrkB (E281A) was specifically expressed in CKII^+^ neurons. Solid outline, dashed outline, arrowheads, and arrows indicate the infected area, non-infected area, CKII^+^ neurons infected with optoTrkB (E281A), CKII^+^ neurons not infected with optoTrkB (E281A), respectively. (**I**–**N**) LED Light stimulated CKII^+^ neurons show higher pERK intensity than unstimulated CKII^+^ neurons. Representative images of V1 neurons stained with antibodies of anti-HA (**I**,**L**), anti-pERK (**J**,**M**), and merged (**K**,**N**). Neurons in stimulated (**L**–**N**) and unstimulated (**I**–**K**) hemispheres. Arrowheads point to CKII^+^-neurons infected with optoTrkB (E281A). Scale bar (**A**,**B**) = 500 um; scale bar (**C**–**N**) = 50 um. (**O**) Regression slope for the effect of LED Light Stimulation on pERK. OptoTrkB (E281A) stimulation significantly increased pERK intensity for CKII^+^ neurons. The regression equation is as follows: (pCREB=2.676+0.640∗LEDstimulation+0.007∗HAintensity+error). * *p* < 0.05. (**P**) LED light stimulation increases optoTrkB (E281A) downstream signalling in CKII^+^ neurons to an equal extent in neurons expressing low (β1=0.761, dotted line), average (β1=0.635, solid line) and high (β1=0.509, dashed line) optoTrkB (E281A) levels.

CKII-Cre mice underwent transparent skull surgery prior to light stimulation. Immunohistochemical analysis of the brains of CKII-Cre mice infected with a pAAV-hSyn1-DIO-OptocytTrkB(E281A)-HA construct verified that they exhibited HA in the visual cortex (Figure 3A–H). Moreover, co-staining of HA and CKII antibodies in individual neurons affirmed that optoTrkB (E281A) was expressed specifically in CKII^+^ neurons (Figure 3C–H).

As with PV^+^ interneurons, a cutoff or minimum HA intensity value was set in order to ensure that only infected neurons were analysed. The distribution of HA intensity in cells from the infected area (N = 107) was compared to those of an uninfected adjacent area (N = 80) (Figure S3). The cutoff point was established in the same way as for PV^+^ interneurons, leaving 50 stimulated neurons and 53 unstimulated neurons for the final analysis (Figure S4).

The intensity of pERK was further analysed in neurons expressing optoTrkB (Figure 3I–N). The raw pERK intensity scores underwent natural logarithmic data transformation to correct for a violation of homoscedasticity. The transformed variable was used as the dependent variable in the data analysis. A multiple regression model for this data indicated an overall significant difference in pERK between the groups (F(3, 99) = 34.002, *p* < 0.001), but no significant interaction effect (β = 0.001, *p* = 0.494) (Figure 3P). After the non-significant interaction term was excluded from the model, a multiple regression assessing the effect of optoTrkB (E281A) stimulation and HA intensity on pERK intensity revealed a significant difference in pERK between the groups (F(2, 100) = 51.039, *p* < 0.001). pERK was significantly higher in the stimulated neurons (β = 0.640, *p* = 0.001) (Figure 3O). These results indicate that optoTrkB (E281A) stimulation increased pERK intensity to a similar extent for neurons expressing low, average, and high levels of optoTrkB (E281A).

## 3. Discussion

This study assesses the activation of a new, highly sensitive, optically activatable optoTrkB (E281A) receptor in PV^+^ interneurons and CKII^+^ pyramidal neurons of the visual cortex. We demonstrate that the highly sensitive optoTrkB (E281A) can be introduced in a specific neuron type in a specific area of interest, and activated using weak, short-term light stimulation in flexible contexts. The activation took place even when optoTrkB (E281A) was stimulated through intact fur and skull.

The analyses of pCREB and pERK intensity show that LED light stimulation results in an increase in downstream signalling of optoTrkB (E281A), both in inhibitory PV^+^ interneurons, as well as in the excitatory CKII^+^ neurons, as shown by a previous study [13]. Interestingly, the higher the expression of optoTrkB (E281A) in PV^+^ interneurons, the more downstream signalling was promoted by light stimulation, while LED light stimulation failed to activate neurons with low levels of optoTrkB (E281A). In contrast, optoTrkB (E281A) activation is the same in CKII^+^ pyramidal neurons expressing low and high levels of optoTrkB (E281A). In other words, LED light stimulation promotes downstream signalling independent of the level of optoTrkB (E281A) in CKII^+^ neurons. There are some possible explanations for this effect. Most likely, transparent skull surgery in the case of CKII^+^ neurons allowed the light to sufficiently stimulate all neurons, including ones expressing low levels of optoTrkB (E281A). Injecting a higher concentration of the AAV9-DIO-optoTrkB (E281A) may allow light stimulation through the intact skull and fur to activate all optoTrkB (E281A) expressing neurons.

Previous research suggests great potential of manipulating TrkB activity for treatments of maladaptive neuropsychiatric symptoms. Recently, our group showed that optoTrkB stimulation in PV^+^ interneurons of the visual cortex shifted ocular dominance by increasing network plasticity [11]. OptoTrkB stimulation decreased PV^+^ interneuron excitability and promoted LTP and oscillatory synchrony in the visual cortex due to the disinhibition of pyramidal neurons [11]. In fact, Donato and colleagues showed that pharmacogenetic inhibition of hippocampal PV^+^ interneurons changed the configuration of PV^+^ interneurons, and improved learning [6]. Our group also showed that optoTrkB stimulation of CKII^+^ pyramidal neurons in the ventral hippocampus increased neural plasticity in the form of increased expression of FosB and potentiation of LTP [17]. In addition, the activation of optoTrkB combined with extinction training reduced fear response in fear-conditioned mice [17]. These findings support the theory that TrkB activation plays a key role in facilitating network plasticity [1,8,9], although the reasons why TrkB activation in both inhibitory and excitatory neurons increase LTP remains unclear. Consequences of promoted neural plasticity by TrkB activation may vary across neuronal subtypes and brain areas, especially in terms of their electrophysiological properties. Further studies are needed to elucidate the different downstream pathways of TrkB activation depending on neuronal subtypes and brain areas.

An interesting avenue for future research is to investigate the effects of increased neural plasticity in specific neural nuclei, where various types of neurons are interacting with each other [18]. For instance, the nucleus accumbens (NAc), a subcortical nucleus in the basal ganglia, has been shown to play a key role in reward, motivation, and drug use [19,20]. A recent review found that individual NAc synapses follow different mechanisms of plasticity [21], and optoTrkB (E281A) can help to illuminate such unique features of neural plasticity as well as aid understanding of the role that plasticity of single neuronal subtypes plays in brain nuclei.

It is known that inhibitory interneurons in the prefrontal cortex (PFC) are involved in several different neuronal plasticity-related behaviours, such as reversal learning [22], extinction of drug addiction [23], and experienced pain [24,25]. This wide array of different behavioural consequences of interneuron plasticity raises questions about what determines the behavioural outcome of interneuron plasticity in the PFC.

The highly sensitive optoTrkB (E281A) allows us to conduct wireless optogenetics and can be applied for high flexibility in different behavioural experimental paradigms, such as social interaction [26,27] and Intellicage [28], where multiple animals have to be simultaneously tested. Thus, optoTrkB (E281A) can be used to explore the behavioural consequences of increased neural plasticity in different neural circuits.

One limitation to note is that this study did not compare transparent and intact skull stimulation of both CKII-Cre and PV-Cre mice. As the sensitivity of optoTrkB (E281A) could differ in CKII^+^ neurons and PV^+^ interneurons, the neuron type used in each stimulation protocol may act as a confounding variable in this study. To eliminate this possibility, future studies should use both PV-Cre and CKII-Cre mice to compare LED light stimulation protocols through a transparent and intact skull.

These limitations notwithstanding, the optoTrkB (E281A) that was evaluated in this study appears sensitive enough to be activated through intact skull and fur. This high sensitivity allows for a lot of flexibility in light stimulation protocols, as no optic cannula or transparent skull is needed to ensure sufficient light intensity entering the brain. Moreover, the highly sensitive optoTrkB (E281A) may be stimulated by using a LED light source on the cage lid, which reduces human handling of the mice and thus the stress that animals experience via direct handling [29]. These features make optoTrkB (E281A) a highly flexible tool to further illuminate the role of TrkB in neuroplasticity.

## 4. Materials and Methods

### 4.1. OptoTrkB DNA

This study used pAAV-hSyn1-DIO-OptocytTrkB(E281A)-HA as previously described [13]. The hSyn1 (Human synapsin 1) promoter allows the virus to target only neurons, not other cell types [30]. DIO codes for double-inverted open reading frame structure. Two incompatible lox-P sites are present on each side of the optoTrkB (E281A) sequence, allowing the transcription of optoTrkB only in Cre-expressing neurons via the Cre-recombinase-mediated inversion of the open reading frame [14,15]. This allows selective expression of optoTrkB (E281A) in defined neuronal subpopulations. Furthermore, cyt refers to the DNA coding for only the cytoplasmic region of the TrkB receptor, which ensures that optoTrkB (E281A) is only stimulated by light and not BDNF-binding. The HA (human influenza hemagglutinin) tag is attached to the C-terminal of the PHR region of optoTrkB (E281A), allowing us to detect optoTrkB (E281A) by immunological methods. The plasmid was packed into AAV serotype 9 (AAV9) by the AAV core unit at the University of Helsinki. AAV9 was selected because it has been shown to allow for stable gene expression in cortical neurons [31,32].

### 4.2. Infection of AAV-OptoTrkB into Mice

AAV9-optoTrkB (E281A) was injected into five mice expressing Cre in CKII^+^ pyramidal neurons, and six mice expressing Cre in PV^+^ interneurons. The visual cortex (V1) was selected as the brain area of interest in this study because it has been extensively studied in iPlasticity studies to date [10,33,34]. For virus injection, the mice were anaesthetised with Isoflurane and fixed on a stereotaxic frame. The virus was injected bilaterally into the visual cortex, 2.8 mm caudally and 2.5 mm laterally from Bregma, at a depth of 0.8 mm. In total, 500 nL of virus solution was injected [13]. After injection, at least four weeks were allowed for expressing optoTrkB (E281A).

Immunohistochemical analysis later revealed that one CKII-Cre mouse had very few cells expressing optoTrkB (E281A) (due to poor sample preparation), and this mouse was excluded from data analysis, reducing the final sample size for CKII-Cre mice from five to four.

### 4.3. LED Light Stimulation

For stimulation of PV-Cre mice injected bilaterally with AAV9-optoTrkB (E281A), the blue light stimulation was applied atop the head with an intact skull and fur. A 488 nm blue LED light pulse was given for one second every five seconds, for a total duration of 30 min. Half of the mice underwent blue LED light stimulation (*n* = 3), while the rest of the mice were exposed to a red LED light as a control group (*n* = 3).

CKII-Cre mice injected bilaterally with AAV9-optoTrkB (E281A) underwent transparent skull surgery in order to ensure sufficient intensity of the blue LED light to pass through the skull (for detailed protocol, see [35]). The transparent skull was covered with black nail polish until light stimulation took place. For optoTrkB-stimulation, a continuous 30-s 488 nm blue LED light was exposed directly onto the transparent skull, atop the right hemisphere. The left hemisphere was left unstimulated and thus functioned as a control condition for each mouse (*n* = 5). The mice were perfused 30 min after light stimulation.

### 4.4. Perfusion

Thirty minutes after the end of light stimulation, the mice were perfused in order to obtain the brains for immunohistochemistry. The mice were anaesthetised using pentobarbital (Mebunat, Orion Pharma, Espoo, Finland) mixed with Lidocaine (Orion Pharma), and the mice were fixed via transcardiac perfusion with 4% paraformaldehyde in pH 7.4 phosphate-buffered saline (PBS) with 3–4 mL per minute for 15–20 min. The brains were then dissected and stored in 4% paraformaldehyde overnight, after which the brains were transferred to 0.04% sodium azide in PBS until cutting. The brains were then embedded in 3% agar gel and cut into 40 μm slices using a vibratome (Leica Biosystems, Deer Park, IL, USA).

### 4.5. Immunohistochemistry

For pCREB immunohistochemistry, antigen retrieval was used to ensure sufficient binding of the primary antibodies to their antigens of interest. Secondly, for pERK immunohistochemistry, Tyramide Signal Amplification (TSA) (Perkin Elmer, Shelton, CT, USA) was used to enhance the signal emitted by pERK as previously described [13]. Briefly, for signal amplification via antigen retrieval, slices from the visual cortex were incubated at 70 °C in 0.01 M citric acid and further treated with 0.1 M Glycine in PBS. The slices were then blocked with 3% bovine serum albumin, 10% normal donkey serum and 10% normal goat serum, to block nonspecific binding of secondary antibodies made in these animals, as well as with Mouse-anti-Mouse buffer (Vector Laboratories, Newark, CA, USA), to block nonspecific binding of antibodies to endogenous mouse Immunoglobulin G. The slices of PV-Cre mice were then successively stained with anti-HA (1:500, Cell Signalling Technology, Danvers, MA, USA, 6E2 #2367), anti-PV (1:1000, Synaptic Systems, Göttingen, Germany, #19500), and anti-pCREB (1:1000, Cell Signalling Technology, #9198) diluted in PBS with 0.3% TritonX for 24–72 h at 4 °C. For CKII-Cre mice, the slices were similarly stained with anti-HA (1:800, Cell Signalling Technology, CST3724), anti-CKII (1:250, Abcam, Cambridge, United Kingdom, #22609), and anti-pERK (1:10,000, Cell Signalling Technology, #9101). All antibodies have been validated by previous studies (see Table S1). After washing in TNT buffer (0.1 M Tris-HCl pH 7.5, 0.15 M NaCl, 0.05% Tween-20) the samples were reacted with secondary antibodies conjugated with fluorophores, or with horseradish peroxidase combined with TSA. A catalogue of the antibodies used is listed in Supplemental Table S1. After washing again with TNT, the samples were transferred to 0.1 M phosphate buffer and mounted onto glass slides with anti-fade mounting medium (Agilent Dako Fluorescence, Santa Clara, CA, USA).

### 4.6. Data Analysis

Image analysis using confocal images was conducted by following previously reported methods [6,36,37,38]. Detailed descriptions of the data analysis can be found in the Supplementary material (Supplementary Note S1). The slices were imaged using the Andor Dragonfly 505 spinning disc confocal microscope. The imaging settings (e.g., pinhole size, laser power, gain, and scan speed) were established with samples and negative controls (not stained with the primary antibody) in order to avoid overexposure and oversaturation, which would limit the dynamic range of the detectors [36,37]. Imaging settings were kept exactly the same for all samples to allow for quantification of emitted fluorescence [36,37]. The images were analysed in ImageJ. Between 10 and 15 infected neurons were selected per image, and intensity values of HA, PV, and pERK or pCREB were recorded. Briefly, the intensity value was quantitatively recorded from each channel by outlining the neuron at its widest point in the z-stack, and the mean brightness value across all pixels within the outlined area was used as the intensity value. To ensure that only infected neurons were included in the final data analysis, a cutoff value of HA intensity was established by measuring HA intensity values from ten uninfected neurons adjacent to the infected area. A more detailed description of the image analysis can be found in the Supplementary material (Note S1).

After the selection of infected neurons was finalised, the change in pERK or pCREB intensity as a result of optoTrkB (E281A) stimulation was analysed with multiple regression and simple slope analysis [39,40]. Analyses were done separately for PV-Cre and CKII-Cre mice. HA intensity as well as an interaction term of HA intensity and stimulation condition was first included in the regression model, and where the interaction term was insignificant, it was removed from the model. When the interaction term was significant, follow-up analysis was conducted using simple slopes analysis with Bonferroni correction. A detailed description of the statistical analysis can be found in the Supplementary Materials (Note S1).

## 5. Conclusions

In this study, we evaluated the utility of a new, highly sensitive, optically activatable TrkB receptor. OptoTrkB (E281A) was successfully transduced in PV^+^ interneurons and CKII^+^ pyramidal neurons specifically. Light stimulation through transparent skulls or even through a high opacity barrier (intact skull and fur) promoted phosphorylation of ERK and CREB, downstream signals of TrkB, in the neurons expressing optoTrkB (E281A) at a certain level. Our results indicate that this highly sensitive optoTrkB (E281A) receptor can be activated using wireless optogenetic methods, and thus can be used for a broad range of behavioural studies, such as social interaction tests and Intellicage. With this tool, we can study the role of TrkB signalling in different neural subtypes in the short and long term, which will help with developing more effective treatments for neuropsychiatric disorders, such as depression, post-traumatic stress disorder, and drug addiction.

## Data Availability

Not applicable.

## Supplementary Materials

The supporting information can be downloaded at: https://www.mdpi.com/article/10.3390/ijms231810249/s1. References [6,11,36,37,39,40,41,42,43,44,45,46] are cited in the supplementary materials.
