# Supplementary material for: Optical Activation of TrkB (E281A) in Excitatory and Inhibitory Neurons of the Mouse Visual Cortex
Source: Int J Mol Sci. 2022 Sep 6;23(18):10249. doi: 10.3390/ijms231810249 (PMC9499497; doi:10.3390/ijms231810249)
Supplement: Supplementary file 1 [file ijms-23-10249-s001.zip › ijms-1869270-supplementary.pdf]

Supplementary materials: Optical Activation of TrkB (E281A) in Excitatory and Inhibitory Neurons of the Mouse Visual Cortex

Antonia Lilja, Giuliano Didio, Jongryul Hong, Won Do Heo, Eero Castrén, Juzoh Umemori

Figure S1

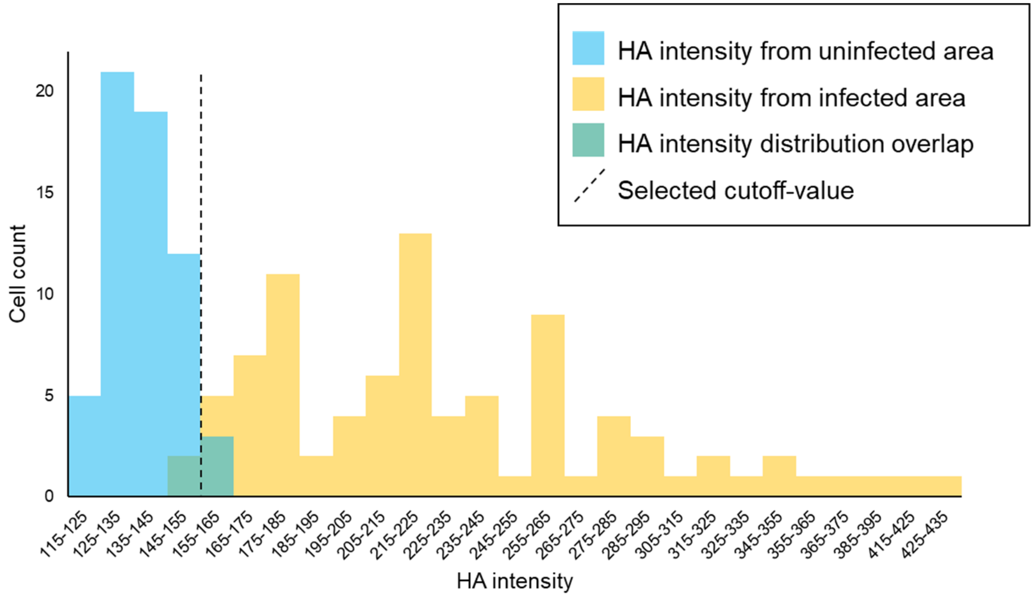

Legend:

HA Intensity Distribution in Infected and Uninfected PV+ Interneurons. The cutoff value was selected such that less than 5% of analysed neurons expressed HA in the range of neurons selected from the uninfected area. Neurons from the infected area expressing HA intensity below the cutoff value were excluded from further analysis.

Figure S2

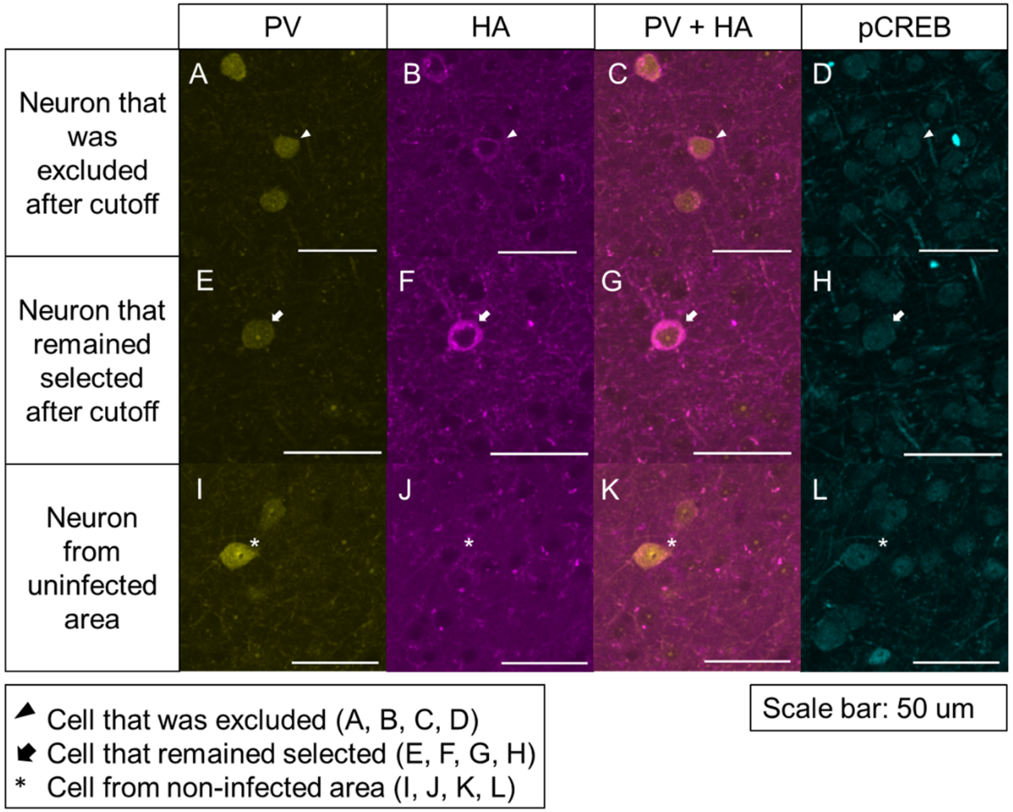

Legend:

PV+ Interneuron Excluded From Analysis Based on HA Cutoff-value in Comparison With Uninfected Neuron and Neuron That Remained Selected. Images from immunohistochemistry of PV-cre mice expressing optoTrkB in PV+ interneurons; (A, E, I) anti-PV, (B, F, J) anti-HA, (C, G, K) merged image of anti-PV and anti-HA, and (D, H, L) anti-pCREB. PV, parvalbumin; HA, human influenza hemagglutinin, tag attached to optoTrkB (E281A); pCREB, phosphorylated CREB.

Figure S3

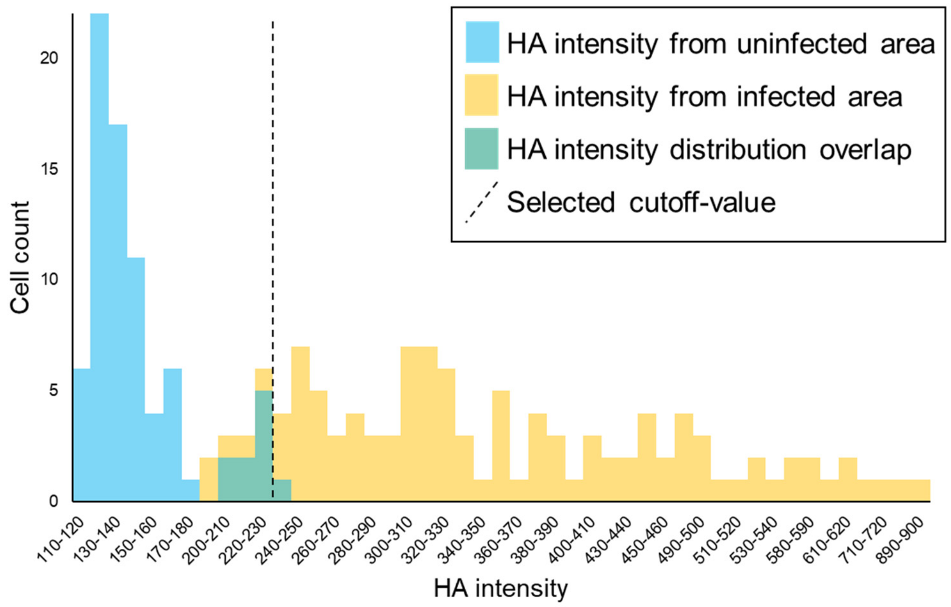

Legend:

HA Intensity Distribution in Infected and Uninfected CKII+ Neurons. The cutoff value was selected such that less than 5% of analysed neurons expressed HA in the range of neurons selected from the uninfected area. Neurons from the infected area expressing HA intensity below the cutoff value were excluded from further analysis.

Figure S4

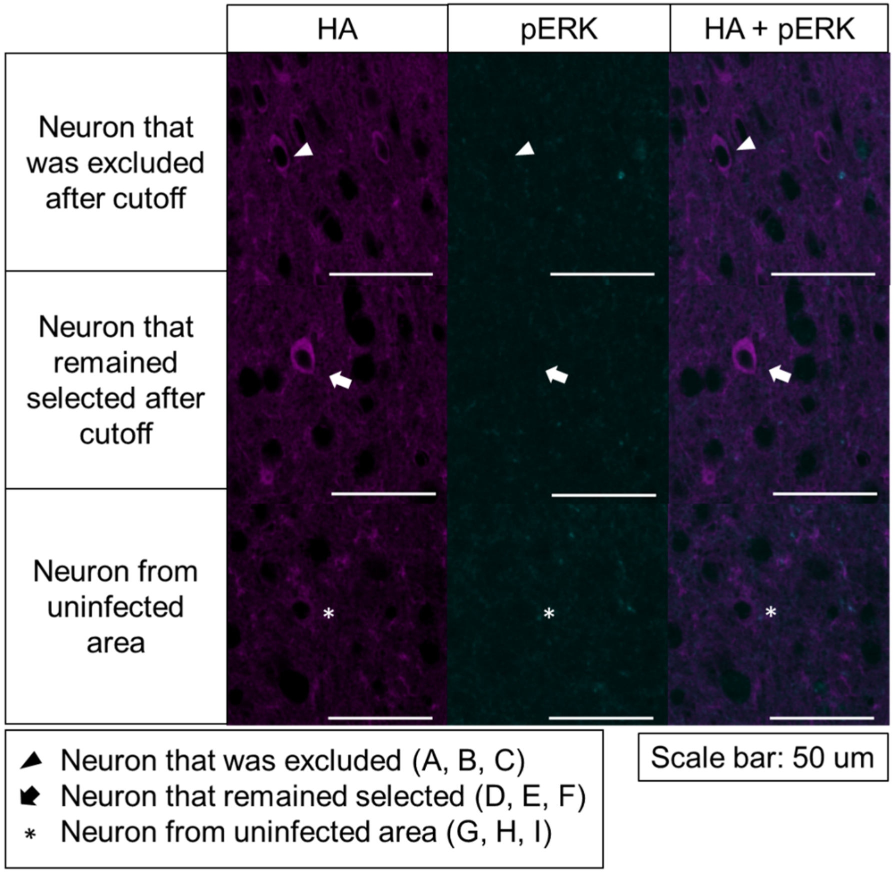

Legend:

Example of Excluded CKII+ Neuron in Comparison With Uninfected Neuron and Neuron That Remained Selected. Images from immunohistochemistry of CKII-cre mice expressing optoTrkB in CKII+ neurons; (A, D, G) anti-HA, (B, E, H) anti-pERK, and (C, F, I) merged image of anti-CKII and anti-pERK. HA, human influenza hemagglutinin, tag attached to optoTrkB (E281A); pERK, phosphorylated ERK.

Table S1

Title: Catalogue of antibodies

	Antibody	Dilution	Company	Validated previously in
**Primary antibodies**	Rabbit anti-HA (CST3724)	1:800	Cell Signalling Technology	[1]
	Mouse anti-HA (6E2, #2367)	1:500	Cell Signalling Technology	[2,3]
	Guineapig anti-PV (19500)	1:1000	Synaptic Systems	[4,5]
	Rabbit anti-pCREB (9198)	1:1000	Abcam	[6]
	Mouse anti-CKII (Ab22609)	1:250	Cell Signalling Technology	[7]
	Rabbit anti-pERK (9101)	1:10000	Cell Signalling Technology	[8]
**Secondary antibodies**	Goat anti-Rabbit conjugated with horseradish peroxidase (1705046)	1:1000	Biorad	
	Donkey anti-Mouse conjugated with Alexa 568	1:400	Invitrogen, ThermoFisher	
	Donkey anti-Guineapig conjugated with Alexa 488	1:400	JacksonImmuno	
	Donkey anti-Rabbit conjugated with Alexa 568	1:400	Invitrogen, ThermoFisher	
	Donkey anti-Mouse conjugated with Alexa 647	1:400	Invitrogen, ThermoFisher	

Note S1: Data analysis

1. Confocal Imaging And Image Analysis

The visual cortex and surrounding areas were imaged using the Andor Dragonfly microscope with a 40x objective and Fusion software. A Z-stack through the whole slice was obtained from each section with 3μm intervals. The imaging settings (e.g. pinhole size, laser power, gain and scan speed) were established with samples and negative controls (not stained with the primary antibody) in order to avoid overexposure and oversaturation, which would limit the dynamic range of the detectors [9,10]. For detailed information about the quantification of confocal images, refer to previously published articles [9,10]. The imaging settings were kept exactly the same for all images.

The images were quantitatively analysed using ImageJ software by following methods reported in previously published papers [5,9,10]. Briefly, HA was used to identify neurons expressing optoTrkB (E281A). The overlap of HA and PV or CKII was observed to assess whether optoTrkB (E281A) was successfully infected in those neuron subpopulations. pCREB or pERK, downstream markers of TrkB activation, were used to evaluate the extent of optoTrkB (E281A) activation and downstream signalling. From each image, 10-15 infected neurons from the visual cortex were selected for analysis. Then each neuron was outlined at its widest point in the z-stack by using the ImageJ selection tool. The intensity value of HA, PV and pCREB or pERK was measured individually for each marker by obtaining the average intensity of fluorescence across all pixels in the selected area. The brightness value in each pixel is computed by ImageJ according to the equation below, where R, G and B indicate the red, green and blue luminosities in the pixel.

$$brightnessvalue = (R + G + B)/3$$

The intensity value of pCREB or pERK was used for comparing optoTrkB (E281A) activation and downstream signalling in stimulated and unstimulated mice or hemispheres. The intensity value of HA was used as a control variable, since the amount of downstream signalling may depend on the extent to which optoTrkB (E281A) is expressed in the neuron.

To ensure that only optoTrkB (E281A) infected cells were selected, ten neurons per mouse were selected from an uninfected area adjacent to the visual cortex. The distribution of HA intensity in cells from this area was compared to the distribution of HA intensity in cells from the infected visual cortex to establish a minimum HA intensity that the visual cortex neuron needed to reach to be considered as expressing optoTrkB (E281A).

2. Multiple regression analysis of the effect of LED light stimulation on optoTrkB (E281A) downstream signalling

The data were analysed using multiple regression and simple slope analysis [11,12]. The distribution of pCREB or pERK intensity was examined before multiple regression analysis, and where the data severely violated the homoscedasticity assumption, a natural log transformation of the original scores was used as the dependent variable. In addition to the light stimulation condition, the centered HA intensity for each cell was included as a predictor. This was done in order to correct for the effect of infection rate on optoTrkB (E281A) activation.

Furthermore, to ensure that only optoTrkB (E281A) infected cells were selected for analysis, a minimum cutoff value of HA intensity was established by comparing the distributions of HA intensity of cells in the infected visual cortex and cells in an adjacent uninfected area. The cutoff value was selected such that less than 5% of the analysed cells had a HA intensity which fell into the range of cells from the uninfected area.

Initially, an interaction term of centered HA intensity and LED light stimulation group was included. When the interaction term was insignificant, it was removed from the regression model. When it was significant, simple slope analysis was used to examine the effect of LED light stimulation at average, low and high HA intensities. The significance of the results was interpreted at $\alpha = .05$. For multiple comparisons, Bonferroni correction was applied.

References

1. Song, A.; Zhu, L.; Gorantla, G.; Berdysz, O.; Amici, S.A.; Guerau-de-Arellano, M.; Madalena, K.M.; Lerch, J.K.; Liu, X.; Quan, N. Salient Type 1 Interleukin 1 Receptor Expression in Peripheral Non-Immune Cells. *Sci Rep* **2018**, *8*, 723, doi:10.1038/s41598-018-19248-7.
2. Field, J.; Nikawa, J.; Broek, D.; MacDonald, B.; Rodgers, L.; Wilson, I.A.; Lerner, R.A.; Wigler, M. Purification of a RAS-Responsive Adenylyl Cyclase Complex from Saccharomyces Cerevisiae by Use of an Epitope Addition Method. *Mol Cell Biol* **1988**, *8*, 2159–2165, doi:10.1128/mcb.8.5.2159-2165.1988.
3. Erondu, N.; Kennedy, M. Regional Distribution of Type II Ca2+/Calmodulin-Dependent Protein Kinase in Rat Brain. *J. Neurosci.* **1985**, *5*, 3270–3277, doi:10.1523/JNEUROSCI.05-12-03270.1985.
4. Winkel, F.; Ryazantseva, M.; Voigt, M.B.; Didio, G.; Lilja, A.; Llach Pou, M.; Steinzeig, A.; Harkki, J.; Englund, J.; Khirug, S.; et al. Pharmacological and Optical Activation of TrkB in Parvalbumin Interneurons Regulate Intrinsic States to Orchestrate Cortical Plasticity. *Mol Psychiatry* **2021**, *26*, 7247–7256, doi:10.1038/s41380-021-01211-0.
5. Donato, F.; Rompani, S.B.; Caroni, P. Parvalbumin-Expressing Basket-Cell Network Plasticity Induced by Experience Regulates Adult Learning. *Nature* **2013**, *504*, 272–276, doi:10.1038/nature12866.
6. Rhee, K.D.; Ruiz, A.; Duncan, J.L.; Hauswirth, W.W.; LaVail, M.M.; Bok, D.; Yang, X.-J. Molecular and Cellular Alterations Induced by Sustained Expression of Ciliary Neurotrophic Factor in a Mouse Model of Retinitis Pigmentosa. *Invest. Ophthalmol. Vis. Sci.* **2007**, *48*, 1389, doi:10.1167/iovs.06-0677.
7. Tan, Y.; Rouse, J.; Zhang, A.; Cariati, S.; Cohen, P.; Comb, M.J. FGF and Stress Regulate CREB and ATF-1 via a Pathway Involving P38 MAP Kinase and MAPKAP Kinase-2. *The EMBO Journal* **1996**, *15*, 4629–4642, doi:10.1002/j.1460-2075.1996.tb00840.x.
8. X. Cai, L.; Tanada, Y.; D. Bello, G.; C. Fleming, J.; F. Alkassis, F.; Ladd, T.; Golde, T.; Koh, J.; Chen, S.; Kasahara, H. Cardiac MLC2 Kinase Is Localized to the Z-Disc and Interacts with α-Actinin2. *Sci Rep* **2019**, *9*, 12580, doi:10.1038/s41598-019-48884-w.

9. North, A.J. Seeing Is Believing? A Beginners' Guide to Practical Pitfalls in Image Acquisition. *Journal of Cell Biology* **2006**, *172*, 9–18, doi:10.1083/jcb.200507103.
10. Shihan, M.H.; Novo, S.G.; Le Marchand, S.J.; Wang, Y.; Duncan, M.K. A Simple Method for Quantitating Confocal Fluorescent Images. *Biochemistry and Biophysics Reports* **2021**, *25*, 100916, doi:10.1016/j.bbrep.2021.100916.
11. Howell, D.C. Chapter 15: Multiple Regression. In *Statistical methods for psychology*; Thomson Wadsworth: Australia : Belmont, CA, 2010 ISBN 978-0-495-59784-1.
12. Preacher, K.J. A Primer on Interaction Effects in Multiple Linear Regression. *Quantpsy.org* 2003.
